# The effects of instability training on balance and jump performance in athletes: A systematic review and meta‐analysis

**DOI:** 10.14814/phy2.70650

**Published:** 2025-11-12

**Authors:** Xiaohan Yin, Qinyan Wu, Xinyao Zhao, Lijun Shi, Li Li

**Affiliations:** ^1^ Sport Science School Beijing Sport University Beijing China; ^2^ Laboratory of Sports Stress and Adaptation of General Administration of Sport Beijing Sport University Beijing China

**Keywords:** balance performance, dynamic balance, jump performance, unstable surface

## Abstract

This study evaluates the effects of instability training on athletes' balance and jump performance. After systematically searching databases such as PubMed, Cochrane Library, and Web of Science, we included 14 randomized controlled trials (RCTs) involving 450 athletes. Our analysis found that instability training significantly improved athletes' balance performance, with notable improvements in the one‐legged standing test (SMD = 0.98, 95% CI = 0.73, 1.22, *p* < 0.01), Y balance test (SMD = 0.67, 95% CI = 0.25, 1.09, *p* < 0.01), and standing stork test (SMD = 0.63, 95% CI = 0.29, 0.97, *p* < 0.01). Regarding jump performance, we observed moderate improvements in the vertical jump and single leg jump but found no significant effects in the countermovement jump. The results indicate that instability training effectively improves balance performance. However, its impact on enhancing jump performance is limited.

## 1 INTRODUCTION

Instability training, or “unstable surface training,” involves exercises on devices like balance boards, BOSU balls, foam pads, or suspension systems that challenge postural control by increasing neuromuscular coordination demands when the base of support is reduced or unstable (Wirth et al., 2017). Compared to traditional resistance training, it elicits continuous postural adjustments, enhancing proprioception, joint stabilization, and movement precision under relatively low external loads (Anderson & Behm, 2005; Behm et al., 2015). These adaptations make it popular in strength and conditioning programs, particularly for sports requiring high dynamic balance and body control, such as gymnastics, skiing, and combat sports.

The theoretical basis of instability training lies in the elevated muscle activation and sensorimotor demands it imposes (Behm et al., 2002; Dong et al., 2023). Unstable environments displace the center of mass and disrupt equilibrium, prompting compensatory actions from trunk and lower limb muscles (Anderson & Behm, 2004). While fewer studies have examined long‐term outcomes, evidence suggests that instability training acutely increases muscle activation and chronically improves trunk strength, core muscle activation output (Cuğ et al., 2012), spinal alignment (Stanforth et al., 1998), balance, and motor control (Blasco et al., 2019). Heitkamp et al. (2001) observed improvements in one‐legged balance, lower‐limb strength, and reductions in inter‐limb asymmetry of muscle strength and activation. However, findings are inconsistent. Some argue it may hinder maximal power development due to reduced force output in unstable settings (Fowles, 2010; Willardson, 2007). For instance, Zemkova et al. (2017) reported higher mean power output during stable conditions, with instability causing a greater reduction in power under fatigue. Behm et al. (2002) noted up to a 70% reduction in leg extensor strength under instability, suggesting a trade‐off between stability and force production.

Despite its growing popularity across athletic, rehabilitative, and general fitness settings—including among youth, older adults, recreational trainees, and trained individuals—the specific performance benefits of instability training remain unclear, particularly whether it primarily improves balance or also enhances lower‐limb jump performance (Anderson & Behm, 2005; Behm & Colado Sanchez, 2013). Both capacities are crucial for athletic performance but rely on distinct neuromuscular mechanisms. Balance reflects the effectiveness of the postural control system in maintaining body stability under various conditions, and better balance has been associated with a reduced risk of injuries in athletes. For example, McGuine and Keene (2006) demonstrated that a structured balance‐training program significantly reduced the incidence of ankle sprains in high school athletes. Meanwhile, jump performance reflects explosive strength and the ability to generate rapid force in the lower limbs (Behm & Colado Sanchez, 2013). Previous reviews focused mainly on non‐athletic populations (Behm et al., 2015), limiting applicability to athletes. This meta‐analysis addresses that gap by evaluating the effects of instability training on balance and jump performance in trained athletes, hypothesizing moderate improvements in both, with greater effects on balance.

## 2 METHODS

### 2.1 Study protocol and registration

The study protocol for this systematic review is registered in the PROSPERO database (CRD42024542314) and meets the Preferred Reporting Items for Systematic Reviews and Meta‐Analyses (PRISMA) extended statement criteria (Page et al., 2021).

### 2.2 Search strategy

Articles published before May 1, 2024, were found using the electronic databases PubMed, Cochrane Library, Web of Science, and China National Knowledge Infrastructure (CNKI). With (non‐stable training OR Unstable Training OR Unstable Resistance Training OR non‐stable resistance training OR unstable support surface OR Non‐Balance Training OR instability training OR instability resistance training) AND (athletes OR players OR sportsman OR sports person OR sportswomen OR Athlete OR Professional Athletes OR Athlete, Professional OR Athletes, Professional OR Professional Athlete OR Elite Athletes OR Athlete, Elite OR Athletes, Elite OR Elite Athlete OR College Athletes OR Athlete, College OR Athletes, College OR College Athlete) as subject terms for Boolean logic searches and traced the references included in the study, a new search was conducted 1 year later, but no new articles were identified for inclusion. The systematic search process was conducted by Xy.Z. and Xh.Y. Any disagreement regarding the included/excluded study was resolved by the third author (Qy.W.).

### 2.3 Inclusion and exclusion criteria

The inclusion criteria were as follows: Randomized controlled trials (RCTs) only; healthy athletes; instability training is the primary intervention and may combine with other types of training, but the effects of instability training must be able to be assessed; either a control group that does not receive the intervention or a control group that receives other training methods; studies must report at least one balance and jump performance outcome; Chinese‐language papers are included only in core journal papers.

### 2.4 Outcome measures

Balance‐related outcomes included the one‐legged standing test, standing stork test, and Y balance test.

One‐legged standing test: assesses static balance, quantified as the duration that a participant can maintain a one‐legged stance without losing equilibrium.

Standing stork test: evaluates static postural control, measured as the maximum time an individual can hold the stork position on one leg while keeping the non‐supporting foot on the knee of the supporting leg.

Y balance test: measures dynamic balance, quantified by the normalized composite reach distance (% of leg length) in three directions—anterior, posteromedial, and posterolateral—reflecting the participant's ability to maintain stability during multidirectional reach tasks.

Jump‐related outcomes included the countermovement jump, vertical jump, and single leg jump.

Countermovement jump: assesses bilateral lower‐limb explosive power, quantified as jump height calculated from displacement using force plates or contact platforms.

Vertical jump: measures vertical jump height, expressed as the maximum height achieved during an upward jump from a standing position.

Single leg jump: expressed as the horizontal jump distance measured from the take‐off line to the landing point.

### 2.5 Data extraction

Two researchers (Xy.Z. and Xh.Y.) independently screened the literature. Any discrepancies were resolved through discussion with a third reviewer (Qy.W.). Extracted data included the first author, year of publication, country, athlete type, sex, sample size, intervention details (type, duration, and frequency), and outcome measures (means, standard deviations, and pre–post changes for each indicator). When outcome data were not reported in tables or the main text, we extracted them from figures using WebPlotDigitizer software (version 4.2). If clarification or additional data were required, we contacted the corresponding authors via email or phone. Studies were excluded if essential data could not be obtained.

### 2.6 Risk of bias assessment

Two reviewers (Xy.Z. and Xh.Y.) independently assessed the risk of bias in randomized controlled trials using ROB2.0 (Revised Cochrane Risk of Bias Tool for Randomized Controlled Trials) (Sterne et al., 2019). Reviewers resolved disagreements through discussion, and if they could not reach a consensus, a third reviewer (Qy.W.) acted as adjudicator to make the final decision.

### 2.7 Statistical analysis

Meta‐analysis was performed using RevMan 5.4.0 software. Effect sizes were calculated based on the mean change from baseline and the standard deviation of the change, which was determined using the following equation (correlation coefficient, Corr = 0.5) (Higgins et al., 2019).

$${\mathrm{SD}}_{\text{change}}=\sqrt{\text{SDpre}2+\text{SDpost}2-\left(2\times \text{Corr}\times \text{SDpre}\times \text{SDpost}\right)}$$

Standardized mean differences (SMDs) were interpreted as small (<0.2), medium (0.2–0.5), or large (≥0.5) effect sizes. Heterogeneity was assessed using the *I* ^2^ statistic, with thresholds of low (<25%), medium (25%–50%), and high (>50%) heterogeneity (Deeks et al., 2019). Fixed‐effects models were applied for low and medium heterogeneity, while random‐effects models were used when heterogeneity was high. Sensitivity analyses and subgroup analyses were conducted for outcomes with high heterogeneity to evaluate the influence of individual studies on the pooled results and to explore potential sources of heterogeneity. If no clear source could be identified, descriptive analyses were used to summarize study characteristics. Mean differences (MDs) were calculated when outcomes were measured using the same tools and units, whereas SMDs were used when different instruments or units were employed. The statistical significance of pooled effect sizes was assessed using *Z*‐tests, with *p* < 0.05 considered significant. For analyses involving more than 10 studies, publication bias was evaluated using funnel plots and Egger's test, and, if bias was detected, the trim‐and‐fill method was applied (Page et al., 2019).

## 3 RESULTS

### 3.1 Included studies

**FIGURE 1:**
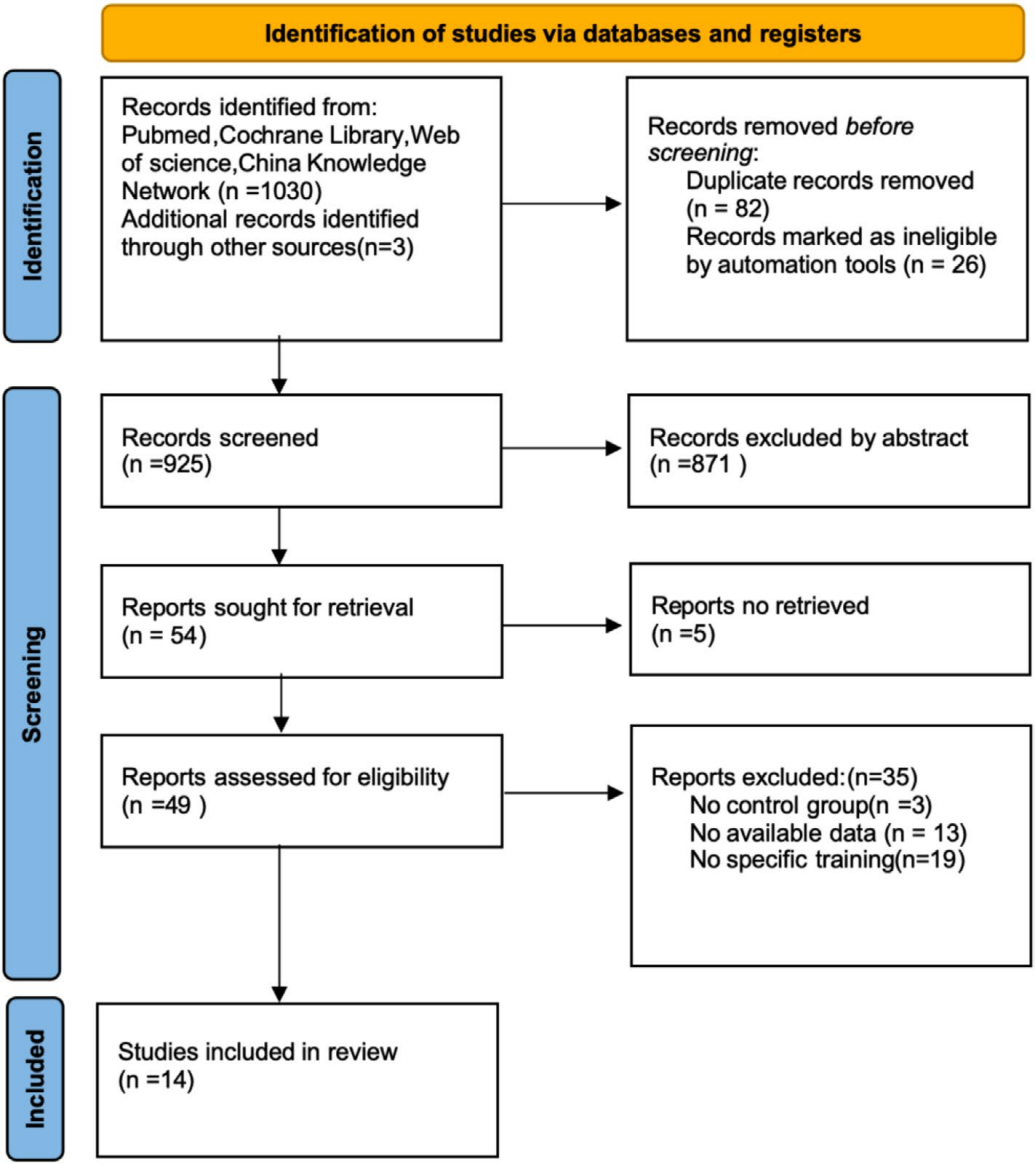
PRISMA flowchart of the study selection process.

A total of 1030 articles were initially identified. After removing 82 duplicates and 26 irrelevant articles, 871 studies were excluded based on title and abstract screening. Five studies were excluded due to unavailable reports. The full texts of 49 studies were reviewed, and 35 were excluded: three lacked a control group, 13 had inappropriate or unanalyzable outcomes, and 19 used interventions not defined as instability training. Additionally, three studies were identified through reference screening. Ultimately, 14 studies were included in the meta‐analysis (Cao, 2022; Cressey et al., 2007; Domeika et al., 2020; Fisek & Agopyan, 2021; Fu & Li, 2014; Gidu et al., 2022; Granacher et al., 2015; Hammami et al., 2022; Lago‐Fuentes et al., 2018; Negra et al., 2017; Prieske et al., 2016; Sanchez‐Sanchez et al., 2022; Sun et al., 2010; Wang et al., 2016). The study selection process is illustrated in Figure 1.

### 3.2 Characteristics of included studies

A total of 14 studies were included (Table 1), published between 2007 and 2022, involving healthy athletes aged 12–23 years. The combined sample size was 453, with 236 in the experimental group and 217 in the control group. Participants represented sports such as tennis, volleyball, football, basketball, handball, and pistol shooting. The experimental group received instability training, while the control group received no intervention, stability training, or traditional functional training. Outcome measures included: One‐legged standing (Fu & Li, 2014; Sun et al., 2010; Wang et al., 2016), Y balance test (Domeika et al., 2020; Fisek & Agopyan, 2021; Hammami et al., 2022), standing stork test (Cao, 2022; Hammami et al., 2022; Negra et al., 2017), countermovement jump (Gidu et al., 2022; Granacher et al., 2015; Hammami et al., 2022; Lago‐Fuentes et al., 2018; Negra et al., 2017; Prieske et al., 2016), vertical jump (Gidu et al., 2022; Hammami et al., 2022), and single‐leg (Gidu et al., 2022; Hammami et al., 2022; Sanchez‐Sanchez et al., 2022). Training duration, frequency, sex, and sport type were also recorded.

**TABLE 1:** Characteristics of included studies.

Author	EG/CON (*n*)	Age		Sex	Sport type	Intervention		Frequency		Outcome	
		EG	CON			EG	CON	EG	CON	EG	CON
Wang et al.	8/10	23.6 ± 3.2	23.3 ± 5.1	F	Pistol	S‐E‐T training	Traditional training	8 weeks, 3 times/week		One‐legged standing	
Fu et al.	8/8	18.50 ± 1.50	18.38 ± 1. 11	M	Tennis	S‐E‐T training	Traditional training	12 weeks, 2 times/week		One‐legged standing	
Sun et al.	8/8	19.25 ± 0.89	19.25 ± 1.28	M	Volleyball	S‐E‐T training	Traditional training	9 weeks, 2–3 times/week		One‐legged standing	
Lago‐Fuentes et al.	7/7	23.8 ± 5.8	23.6 ± 4.8	F	Football	Unstable core training+regular futsal training	Stable core training+regular futsal training	6 weeks, 10.3 ± 0.9 h/week		CMJ	
Fisek et al.	13/12	15.57 ± 0.52	15.63 ± 0.51	M	Basketball	Multi‐dimensional balance training+regular resistance training	Stable surface training+regular resistance training	6 weeks, 2 times/week		YBT	
Cao et al.	12/12	18–21		M	Tennis	Unstable hitting and footwork drills on a balance board	Stabilizing surface footwork and hitting drills	8 weeks, 3 times/week		SST	
Prieske et al.	18/19	16.6 ± 1.0	16.6 ± 1.1	M	Football	CSTU+football training	CSTS+football training	9 weeks, 2–3 times/week		CMJ	
Sanchez‐Sanchez et al.	27/28	18.0 ± 0.4	17.9 ± 0.6	M	Football	Regular resistance training on BOSU	Regular resistance training	10 weeks, 2 times/week		SLJ	
Granacher et al.	12/12	15.2 ± 0.5	15.6 ± 0.6	M	Football	Jump exercises on highly unstable surfaces	Jump exercises on stable surfaces	8 weeks, 2 times/week		CMJ	
Gidu et al.	48/48	14.2 ± 0.4	14.0 ± 0.0	M	Football	Proprioceptive training with bosu ball	Physical training and soccer training	8 weeks, 4 times/week		CMJ, VJ, SLJ	
Cressey et al.	10/9	18 to 23		M	Football	Lower‐body on unstable surface training	Lower‐body on stable surfaces	10 weeks		CMJ	
Domeika et al.	17/14	21.35 ± 0.605		M	Basketball	Abili Balance Trainer	Regular resistance training	8 weeks, 3 times/week		YBT	
Hammami et al.	24/18	16.4 ± 0.4	16.2 ± 0.4	M	Handball	Jump and sprint exercise training on sand	Technical‐tactical training	7 weeks, 3 times/week, 35 min/session		VJ, CMJ, YBT, SLJ, SST	
Negra et al.	16/17	12.4 ± 0.6	12.3 ± 0.5	M	Football	Combined PT on stable and unstable surfaces	PT on stable surfaces	8 weeks, 2 times/week, 25–30 min/session		CMJ, SST	

Abbreviations: CMJ, countermovement jump; CON, control group; CSTS, core strength training performed on stable surfaces; CSTU, core strength training performed on unstable surfaces; EG, experimental group; F, female; M, male; PT, plyometric training; S‐E‐T, sling exercise training; SLJ, single leg jump; SST, standing stork test; VJ, vertical jump; YBT, Y balance test.

### 3.3 Results of the risk of biases

**FIGURE 2:**
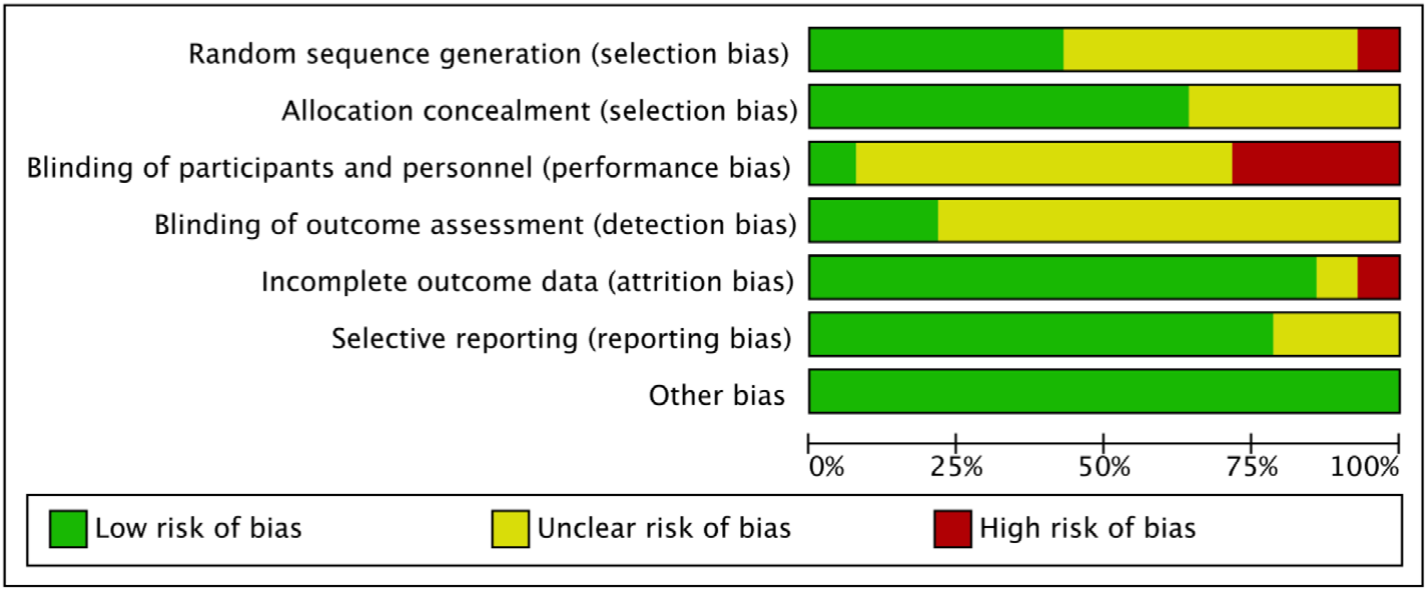
Risk of bias for included studies.

**FIGURE 3:**
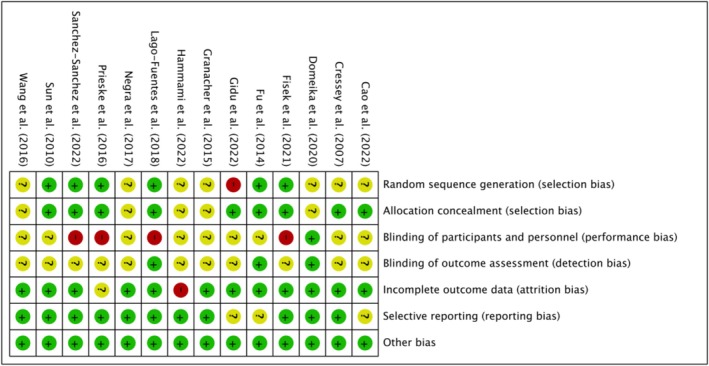
The concrete bias of each study.

All included studies were randomized controlled trials and assessed using the RoB 2.0 tool. Overall results are shown in Figure 2, with domain‐specific assessments in Figure 3. Due to the visible nature of training interventions, blinding of participants and personnel was not feasible, contributing to performance bias. Additionally, pre‐ and post‐intervention physical assessments were inherently observable. As a result, some concerns were noted in Domains 1–3 across several RCTs, due to single‐blind designs, intragroup exercise differences, or participant attrition. Across the 14 studies, a low risk of bias was found in domains related to attrition and other sources. Regarding selection bias, eight studies were rated low risk, one high risk, and five unclear. For reporting bias, 10 studies were rated low risk, and four were unclear.

### 3.4 Synthesis of results

We assessed six performance indicators in athletes, including core stability measures: One‐legged standing, standing stork test, and Y balance test and lower limb muscle strength measures: Countermovement jump, vertical jump, and single leg jump.

**FIGURE 4:**
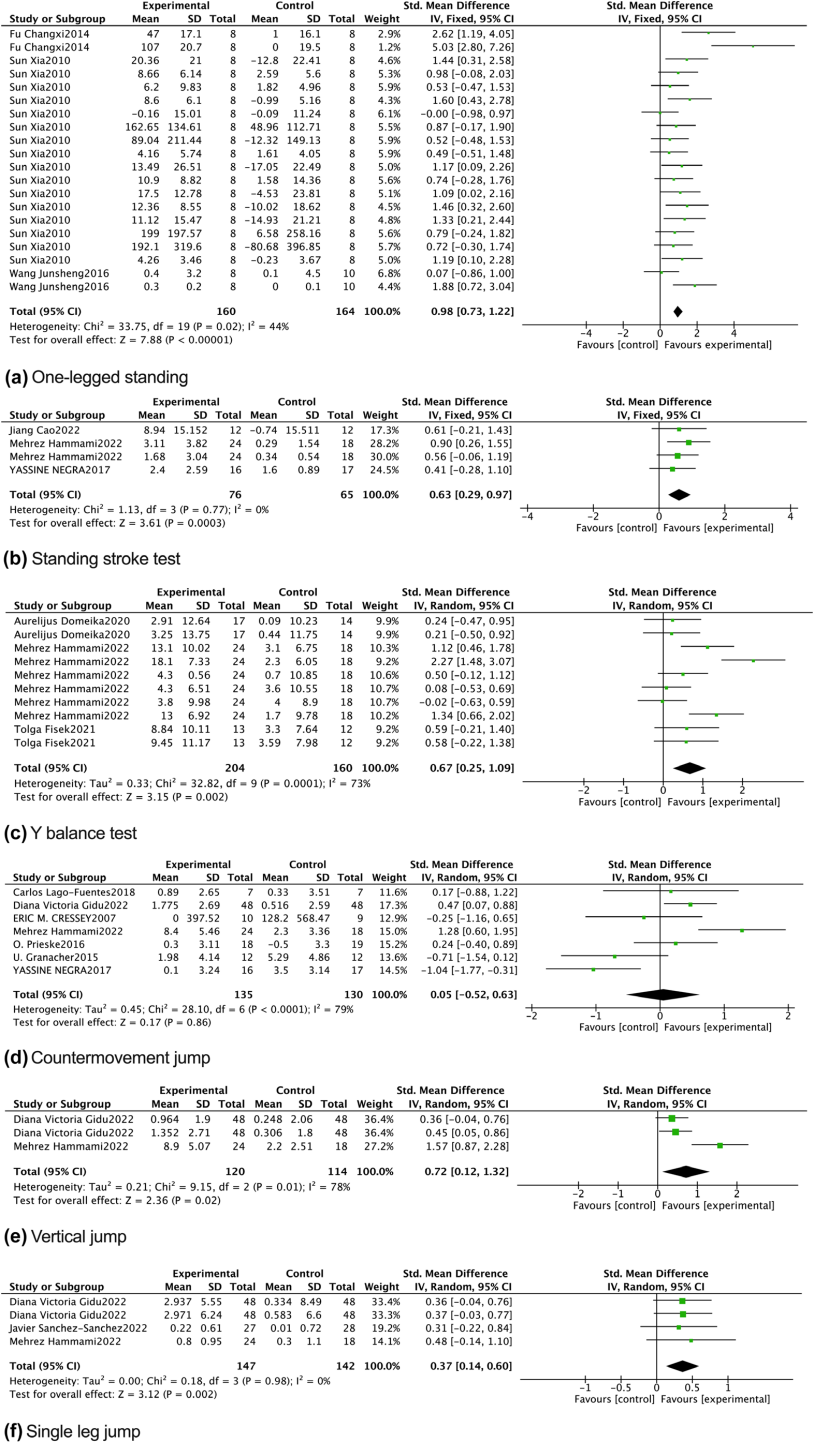
Forest plot.

For one‐legged standing, a meta‐analysis revealed a significantly greater pooled effect size in the experimental group compared to the control group (*p* < 0.01; SMD = 0.98 [0.73, 1.22], 95% CI; *I* ^2^ = 44%) (Figure 4a). This suggests that instability training significantly enhances one‐legged standing performance. Similarly, a meta‐analysis of four studies for the standing stork test showed a significantly higher pooled effect size in the experimental group (*p* < 0.01; SMD = 0.63 [0.29, 0.97], 95% CI; *I* ^2^ = 0%) (Figure 4b), indicating that instability training effectively improves standing stork test performance duration. Combined results from 10 studies demonstrated that instability training had a significant impact on the Y balance test (*p* < 0.01; SMD = 0.67 [0.25, 1.09], 95% CI), though with high statistical heterogeneity (*I* ^2^ = 73%) (Figure 4c).

**FIGURE 5:**
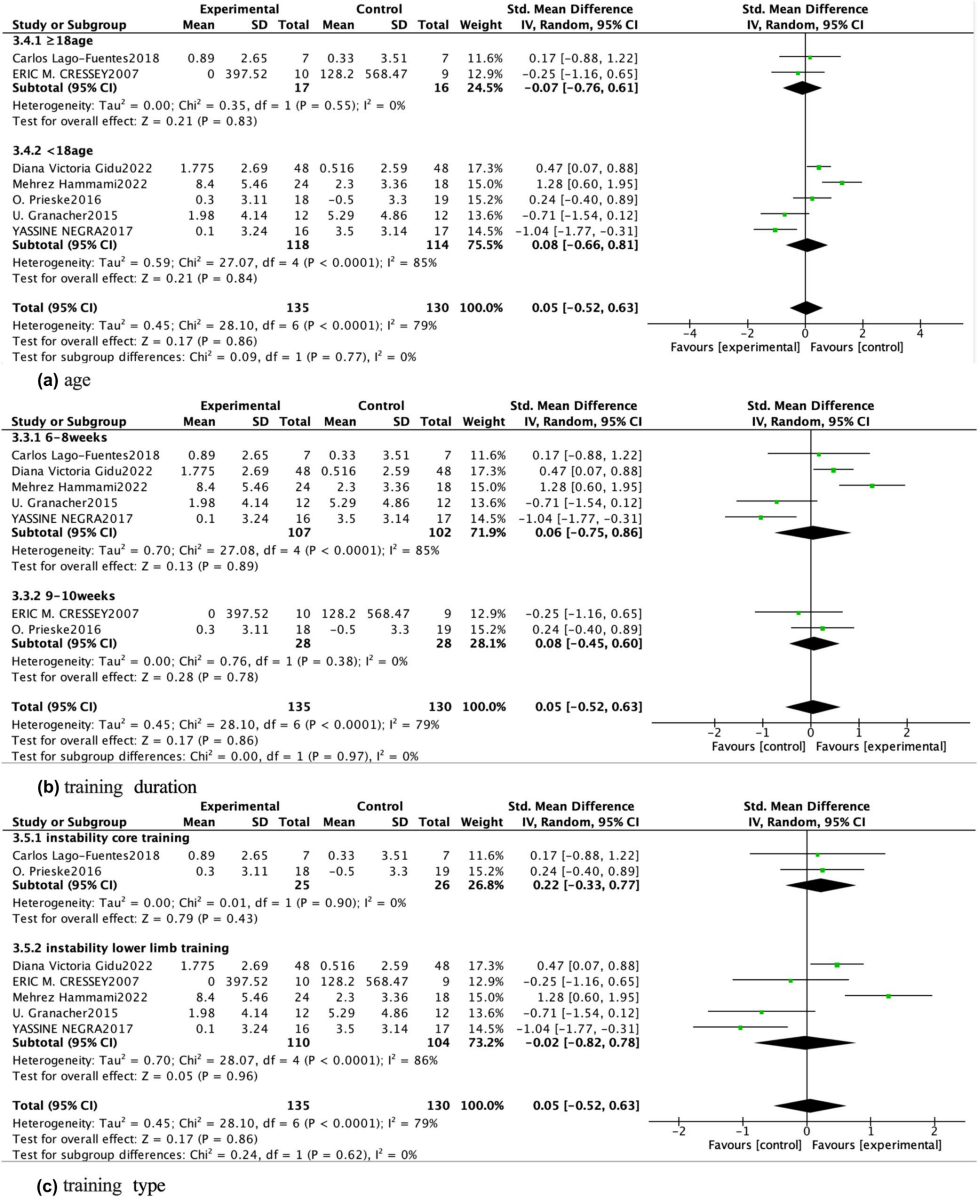
Subgroup analysis.

For countermovement jump, a meta‐analysis of seven studies indicated a small pooled effect size with high heterogeneity (*p* = 0.86; SMD = 0.05 [−0.52, 0.63], 95% CI; *I* ^2^ = 79%) (Figure 4d), suggesting that instability training offers similar effects to traditional stable training for improving countermovement jump height. Subgroup analyses based on age, training duration, and training type were conducted to explore potential sources of heterogeneity in countermovement jump outcomes. However, no significant moderating factors were identified (*p* > 0.05), indicating that these variables had a limited impact on countermovement jump performance (Figure 5). The vertical jump results, derived from three studies, showed a relatively high pooled effect size (*p* < 0.05; SMD = 0.72 [0.12, 1.32], 95% CI) with considerable heterogeneity (*I* ^2^ = 78%) (Figure 4e), indicating that instability training significantly improves vertical jump scores. Finally, combined results from four studies showed a moderate and significant effect of instability training on single leg jump performance (*p* < 0.01; SMD = 0.37 [0.14, 0.60], 95% CI; *I* ^2^ = 0%) (Figure 4f).

### 3.5 Publication of risk of bias analysis

**FIGURE 6:**
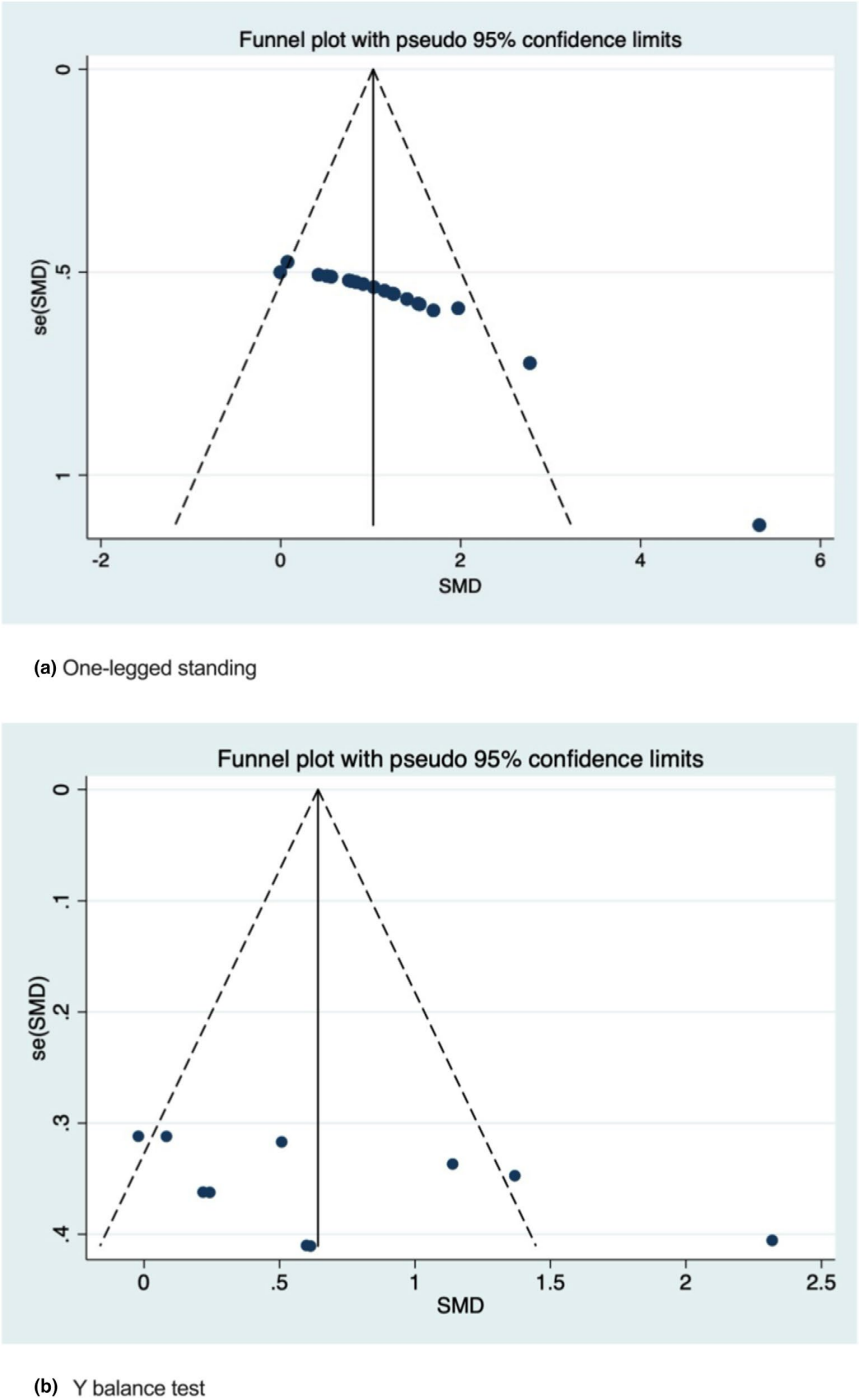
Funnel plot.

**FIGURE 7:**
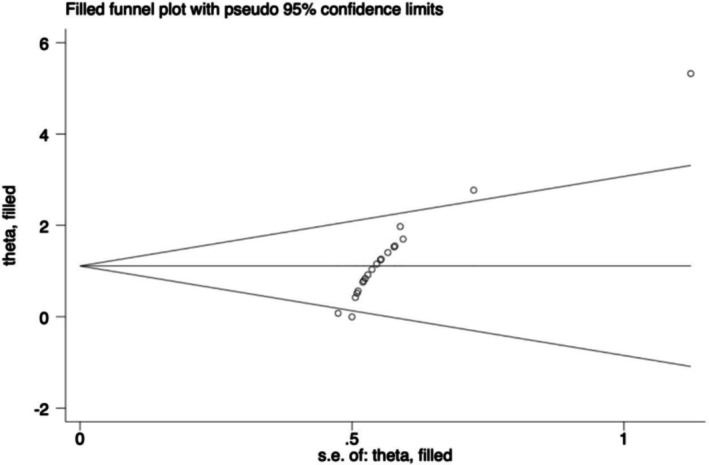
Trim‐and‐fill plot.

In this study, publication bias for the one‐legged standing and Y balance tests was assessed, as both included more than 10 studies. The funnel plot for one‐legged standing (Figure 6) showed asymmetry, and Egger's test indicated significant publication bias (*p* < 0.05). However, the trim‐and‐fill analysis confirmed no changes to the effect size estimations (Figure 7). In contrast, the funnel plot for the Y balance test (Figure 6) also showed asymmetry, but Egger's test did not detect significant publication bias (*p* = 0.161). The detailed results of Egger's test are provided in the Appendix S1.

## 4 DISCUSSION

This meta‐analysis evaluated the effects of instability training on balance and jump performance in athletes across 14 RCTs. Balance outcomes—assessed via the one‐legged standing test, Y balance test, and standing stork test—showed significant improvements in the instability training group. In contrast, the effects on jump performance, including the countermovement jump, were limited. These findings indicate that instability training improves balance as an expression of enhanced postural control, whereas its effects on lower‐limb explosive performance remain limited.

While significant improvements were observed in balance outcomes, high heterogeneity was noted for the Y balance test (*I* ^2^ = 73%). As only three studies reported this outcome, subgroup analysis was not feasible. A sensitivity analysis identified the study by Hammami et al. (2022) as the primary source of heterogeneity. Removing this study reduced heterogeneity to *I* ^2^ = 0% without altering the significance of the pooled effect.

Hammami et al. (2022) incorporated jump and sprint training on sand, introducing multidirectional instability that likely increased postural demands and dynamic balance adjustments, leading to greater improvements in the Y‐balance test. In contrast, Domeika et al. (2020) used rigid, single‐plane platforms that primarily challenged anterior–posterior balance, with limited multidirectional stimulation. Similarly, studies have shown that training on unstable surfaces such as sand can enhance lower‐limb stabilizing capacity and dynamic balance. For instance, Fernández‐Fernández et al. (2023) reported that 6 weeks of neuromuscular training on sand significantly improved dynamic balance and hip adduction/abduction strength in youth tennis players, with greater training load and muscle soreness than training on hard surfaces. These findings suggest that variability in training surfaces and intervention types—particularly between multidirectional (e.g., sand) and unidirectional (e.g., rigid platforms) instability—may explain the heterogeneity observed in Y‐balance outcomes.

The one‐legged standing and standing stork tests primarily assess static balance, reflecting the ability of the postural control system to maintain stability under stationary conditions (Anderson & Behm, 2005). Rigid or single‐plane instability training appears effective for enhancing static balance by improving neuromuscular coordination and joint stability, particularly through the activation of the transverse abdominis and hip stabilizers (Batista et al., 2024; Luo et al., 2023). These adaptations likely improve postural control in the anterior–posterior direction. Instability training enhances both static and dynamic balance primarily through neuromuscular regulation rather than strength gains (Behm et al., 2010). The unstable environment promotes continuous postural adjustments, sustained activation of deep muscles, and enhanced neural responsiveness (Behm et al., 2015; Markovic et al., 2004), including agonist–antagonist coactivation critical for joint stabilization.

Regarding jump performance, moderate improvements were observed in vertical jump and single leg jump, while countermovement jump showed no significant effect. Some heterogeneity was noted in the countermovement jump and vertical jump analysis.

The countermovement jump primarily evaluates bilateral lower‐limb explosive power through coordinated hip, knee, and ankle extension, relying on rapid quadriceps and hamstring activation with minimal balance or neuromuscular coordination involvement. Thus, instability training has limited impact on countermovement jump performance (Lesinski et al., 2018). Drinkwater et al. (2007) demonstrated that performing loaded squats on unstable surfaces such as foam pads or BOSU balls significantly reduced concentric and eccentric power output as well as movement depth, likely due to decreased activation efficiency of the primary force‐producing muscles under unstable conditions. Similarly, Lesinski et al. (2018) reported that postural demands during instability training may delay force production, limiting jump height gains.

To explore heterogeneity sources in countermovement jump outcomes, a multidimensional subgroup analysis was conducted based on participant age, intervention type, and training duration. For participant age, studies were divided into adolescent (≤18 years) and young adult groups (>18 years), and no significant subgroup interaction was found (X^2^ = 0.17, df = 1, *p* = 0.68). For intervention type, studies were categorized as balance‐focused versus jump‐specific instability training, with no significant subgroup difference detected (X^2^ = 0.09, df = 1, *p* = 0.77). Likewise, when stratified by training duration (<8 weeks vs. ≥8 weeks), no subgroup effect emerged (X^2^ = 0.12, df = 1, *p* = 0.73). These findings indicate that the observed between‐study variability cannot be explained by these subgroup factors. Differences in instability equipment across studies—such as sand‐based surfaces versus rigid balance platforms—may represent a more likely source of heterogeneity, although these variables were not consistently reported or quantifiable. Sensitivity analysis further confirmed that excluding individual studies did not substantially alter the overall effect size, which remained stable. Despite this heterogeneity, the findings consistently indicate that instability training has limited efficacy in improving countermovement jump performance.

In contrast to the countermovement jump, the vertical jump, and single leg jump involve distinct movement patterns requiring greater lower‐limb explosive strength, neuromuscular coordination, and dynamic balance control. The Single leg demands unilateral postural stability and effective force transfer to the supporting leg, aligning well with the neuromuscular demands targeted by instability training (Zemkova et al., 2017). Behm and Colado (2012) noted that instability training enhances balance performance during single‐leg support by activating deep stabilizing muscles, leading to improved postural control and performance in unilateral tasks. Similarly, the vertical jump requires multidirectional force coordination and rapid postural adjustments during takeoff and flight. Anderson and Behm (2005) found that improved postural control minimizes body sway, enhancing force transmission, and maximizing jump height. McGuine and Keene (2006) further observed that instability training improves athletes' adaptation to multidirectional force demands, particularly benefiting vertical jump performance.

The meta‐analysis results for the vertical jump showed high heterogeneity, but with only two studies available, subgroup or sensitivity analyses were not feasible. Descriptive analysis suggested that heterogeneity likely stemmed from differences in training methods, participant characteristics, and intervention protocols. Gidu et al. (2022) employed proprioceptive training with foam pads and BOSU balls to enhance dynamic balance and neuromuscular coordination, whereas Hammami et al. (2022) used sand‐based jump training to emphasize lower‐limb explosive strength. Variations in participant age (14 vs. 16–17 years), sport (soccer vs. handball), training frequency (4 vs. 3 sessions per week), and duration further contributed to these differences. These factors likely influenced the specific performance adaptations observed across studies.

Our findings confirm that instability training significantly enhances balance performance, though heterogeneity likely arises from variations in training methods. Low‐instability tools (e.g., balance boards and foam pads) tend to improve static balance, whereas high‐instability methods (e.g., BOSU balls and sand training) more effectively enhance dynamic balance (Albiol‐Pérez et al., 2017). This partly explains variability in Y‐balance test results. High‐instability training demands greater neuromuscular coordination to maintain posture, which may limit maximal force output, affecting countermovement, and vertical jump outcomes (Behm et al., 2010).

The inclusion of only RCTs in this meta‐analysis strengthens the reliability of causal inferences, while focusing on athletes enhances the representativeness of the findings. However, the study inherits limitations common to meta‐analyses and the individual studies included. Heterogeneity in study designs and intervention methods, complicates result interpretation. Additionally, small sample sizes in some studies may have introduced bias, as certain studies carried disproportionate weight in effect size estimation. Finally, the impact of varying degrees of instability on athletes was not assessed, representing an area for future research.

## 5 CONCLUSIONS

This meta‐analysis indicates that instability training can significantly improve balance performance, with limited effects on jump performance. While improvements in postural control are evident, the benefits for lower‐limb power‐related tasks such as the countermovement jump remain modest. For practical application, coaches should align instability training with specific performance goals: traditional high‐load strength training remains more effective for enhancing lower‐limb power, whereas instability training is better suited for activities requiring balance regulation, core control, and multidirectional stability. It may serve as a complementary strategy to support balance‐focused performance, especially in sports with high postural demands. Combining instability training with conventional strength protocols may offer broader benefits. Future research should determine optimal training parameters tailored to specific sports and athlete populations. Furthermore, instability training shows potential as a tool for injury prevention by improving postural control and reducing the risk of maladaptive movement patterns.

## AUTHOR CONTRIBUTIONS

Xiaohan Yin, Li Li, and Lijun Shi contributed to the research concept and study design; Xiaohan Yin and Xinyao Zhao contributed to the literature review, data collection, data analysis, and interpretation; Xiaohan Yin and Qinyan Wu contributed to the statistical analyses and writing of the manuscript; Li Li and Lijun Shi contributed to the reviewing of the manuscript.

## FUNDING INFORMATION

No sources of funding were used to assist in the preparation of this article.

## CONFLICT OF INTEREST STATEMENT

No potential conflict of interest was reported by the authors.

## ETHICS STATEMENT

This meta‐analysis involved the collection of data from previously published studies, all of which were conducted in accordance with the ethical standards outlined by their respective institutional review boards. Given the nature of the study, no new human subjects were involved, and informed consent was not required. The authors declare no conflicts of interest related to this study.

## Supporting information


Appendix S1.

## Data Availability

The datasets generated during and/or analyzed during the current study are available from the corresponding author on reasonable request.
